# Predictors of Progression and Mortality in Patients with Prevalent Rheumatoid Arthritis and Interstitial Lung Disease: A Prospective Cohort Study

**DOI:** 10.3390/jcm10040874

**Published:** 2021-02-20

**Authors:** Natalia Mena-Vázquez, Marta Rojas-Gimenez, Carmen María Romero-Barco, Sara Manrique-Arija, Espildora Francisco, María Carmen Aguilar-Hurtado, Isabel Añón-Oñate, Lorena Pérez-Albaladejo, Rafaela Ortega-Castro, Francisco Javier Godoy-Navarrete, Inmaculada Ureña-Garnica, Maria Luisa Velloso-Feijoo, Rocio Redondo-Rodriguez, Francisco Gabriel Jimenez-Núñez, Blanca Panero Lamothe, María Isabel Padin-Martín, Antonio Fernández-Nebro

**Affiliations:** 1 Instituto de Investigación Biomédica de Málaga (IBIMA), 29010 Málaga, Spain; menchu01@hotmail.com (C.M.R.-B.); fjgodoynavarrete@gmail.com (F.J.G.-N.); inuregar@gmail.com (I.U.-G.); rocioredondo91@hotmail.com (R.R.-R.); cortesfra@gmail.com (F.G.J.-N.); afnebro@gmail.com (A.F.-N.); 2 UGC de Reumatología, Hospital Regional Universitario de Málaga, 29009 Málaga, Spain; 3 Instituto Maimónides de Investigación Biomédica de Córdoba (IMIBIC), 14004 Córdoba, Spain; rojasgimenezm@gmail.com (M.R.-G.); orcam84@hotmail.com (R.O.-C.); 4 UGC de Reumatología, Hospital Universitario Reina Sofía de Córdoba, 14004 Córdoba, Spain; 5 UGC de Reumatología, Hospital Clínico Universitario Virgen de la Victoria, 29010 Málaga, Spain; blanca.panero@gmail.com; 6 UGC de Neumología, Hospital Regional Universitario de Málaga, 29009 Málaga, Spain; fespildorahernandez@gmail.com; 7 UGC de Radiodiagnóstico, Hospital Regional Universitario de Málaga, 29009 Málaga, Spain; maguh007@gmail.com (M.C.A.-H.); maribelpadin@hotmail.com (M.I.P.-M.); 8 Hospital Universitario de Jaén, 23007 Jaén, Spain; isaanononate@gmail.com; 9 Hospital Universitario Virgen de las Nieves, 18170 Granada, Spain; lorenaperezalba@gmail.com; 10 Hospital Universitario Virgen de Valme, 41014 Sevilla, Spain; mlvelloso@hotmail.com; 11 Departamento de Medicina, Universidad de Málaga, 29010 Málaga, Spain

**Keywords:** rheumatoid arthritis, interstitial lung disease, biologics, non-anti-TNF biologics

## Abstract

Objectives: To describe a prospective cohort of patients with rheumatoid arthritis associated with interstitial lung disease (RA-ILD) and identify risk factors associated with disease progression and mortality in this cohort. Patients and methods: We performed a multicenter, prospective, observational study of patients with RA-ILD receiving disease-modifying antirheumatic drugs (DMARDs) between 2015 and 2020. The patients were assessed using high-resolution computed tomography and pulmonary function tests at baseline and at 60 months. The main endpoint was “Progression to ILD at the end of follow-up” in terms of the following outcomes: (1) improvement (i.e., improvement in forced vital capacity (FVC) ≥10% or diffusing capacity of the lungs for carbon monoxide (DLCO) ≥15% and absence of radiological progression); (2) nonprogression (stabilization or improvement in FVC ≤10% or diffusing capacity of the lungs for carbon monoxide (DLCO) <15% and absence of radiological progression); (3) progression (worsening of FVC >10% or DLCO >15% and radiological progression); or (4) death. We recorded demographic and clinical characteristics, lung function, and the incidence of adverse events. A Cox regression analysis was performed to identify factors associated with the worsening of ILD. Results: After 60 months, lung disease had stabilized in 66 patients (56.9%), improved in 9 (7.8%), and worsened in 23 (19.8%). Eighteen patients (15.5%) died, with a mean survival of 71.8 (1.9) months after diagnosis of ILD. The Cox multivariate analysis revealed the independent predictors of worsening of RA-ILD to be usual interstitial pneumonia (hazard ratio (HR), 2.6 (95%CI, 1.0–6.7)), FVC <80% (HR, 3.8 (95%CI, 1.5–6.7)), anticitrullinated protein antibody titers (HR, 2.8 (95%CI, 1.1–6.8)), smoking (HR, 2.5 (95%CI, 1.1–6.2)), and treatment with abatacept, tocilizumab, or rituximab (HR, 0.4 (95%CI, 0.2–0.8)). During follow-up, 79 patients (68%) experienced an adverse event, mostly infection (61%). Infection was fatal in 10/18 patients (55.5%) during follow-up. Conclusions: Lung function is stable in most patients with RA-ILD receiving treatment with disease-modifying anti-rheumatic drugs (DMARDs), although one-third worsened or died. Identifying factors associated with worsening in RA-ILD is important for clinical management.

## 1. Introduction

Interstitial lung disease (ILD) is the most frequent pulmonary manifestation in rheumatoid arthritis (RA), with an incidence of 4 to 4.5/1000 patient-years. The prevalence of RA-ILD varies according to the detection methods used and cohort studied. Although clinically evident ILD occurs in approximately 10% of RA patients, recent studies using high-resolution computed tomography (HRCT) reported a prevalence of 27–67%, with a large percentage of asymptomatic patients. In addition, ILD leads to increased morbidity and mortality and is currently the second most common cause of death in patients with RA after cardiovascular disease [1].

Several studies have attempted to identify factors that help predict poorer prognosis and/or greater mortality in patients with RA-ILD. Those associated with poorer prognosis include advanced age [2,3,4,5,6,7], male sex [3,5,8,9], and factors related to RA itself, such as greater disease duration, autoantibody levels, and poorer control of inflammation [5,10]. Other factors associated with more marked disease progression and mortality in RA-ILD include reduced forced vital capacity (FVC) and diffusing capacity of the lungs for carbon monoxide (DLCO) [11,12] and the radiological pattern of usual interstitial pneumonia (UIP) in high-resolution computed tomography (HRCT) [7,10,13,14,15,16,17,18,19,20].

Despite numerous advances in immunosuppressive therapy, antifibrotic agents, and disease-modifying antirheumatic drugs (DMARDs), data on the effectiveness and safety of these treatments for this challenging condition remain scarce. While some immunosuppressants, such as mycophenolate mofetil, azathioprine, and cyclophosphamide have proven beneficial in ILD associated with systemic autoimmune disease, including RA, these drugs are of little use for joint involvement [21,22,23]. Similarly, antifibrotic agents such as nintedanib could be beneficial only with respect to lung involvement in patients with RA-ILD, as shown in the INBUILD trial [24]. Whereas older studies associated methotrexate with ILD, more recent papers indicate that methotrexate does not appear to be associated with a greater risk [25,26]. Evidence for other conventional synthetic DMARDs (csDMARDs) is rarer, although one meta-analysis did not find a greater frequency of respiratory adverse events with leflunomide [27]. As for biologic DMARDs (bDMARDs), available evidence, which is based mainly on cross-sectional or retrospective studies, suggests that rituximab, abatacept, and tocilizumab could prove safe for treatment of RA-ILD [28,29,30,31,32], whereas tumor necrosis factor inhibitors (anti-TNF) have been associated with a risk of lung impairment [27]. Our group found that non-anti-TNF bDMARDs were associated with poorer short-term progression of lung disease in a prospective cohort of 70 patients with RA-ILD [33]. Given the scarcity of data on the long-term efficacy and safety of these treatments in patients with RA-ILD, the objectives of our study were to describe a prospective cohort of patients with rheumatoid arthritis associated with interstitial lung disease (RA-ILD) and identify risk factors that could help predict prognosis and mortality in these patients in the medium term.

## 2. Patients and Methods

### 2.1. Design

We performed a multicenter observational prospective study of a prevalent cohort of patients with RA-ILD from 6 teaching hospitals in Andalusia, Spain. Recruitment ran from March 2015 to December 2020. The study was approved by the Research Ethics Committee of Hospital Regional Universitario de Málaga (HRUM) (Code 1719-N-15). All of the patients provided their written informed consent before participating in the study.

### 2.2. Study Population

We consecutively recruited adults with RA classified as per the 2010 criteria of the ACR/EULAR [34] and ILD confirmed by means of pulmonary function testing (PFT) and HRCT or lung biopsy. Time since diagnosis of ILD differed when patients were included in the study, and all patients had been receiving a DMARD. We excluded patients who were pregnant and patients with inflammatory or rheumatic diseases other than RA (except secondary Sjögren syndrome), infection, primary pulmonary hypertension, congestive heart failure, and known exposure to fibrosing environmental agents.

### 2.3. Protocol

Selected patients were seen by a rheumatologist who followed a pre-established protocol for clinical and laboratory data collection at the inclusion date (v0), 24 months (v24) [33], and 60 months (v60). PFT and HRCT were performed at v0, v24, v60, and at any other visit if the patients showed symptoms of respiratory impairment or at the attending physician’s discretion. All HRCT scans were performed using an axial 1.5-mm or 2-mm slice at intervals of 1 cm along the thorax. Images were reconstructed using a high spatial frequency algorithm, with an acquisition of 20–25 slices per patient. In order to homogenize the interpretation of findings, the radiological evaluation was centralized at HRUM and performed blind and independently by 2 experts in pulmonary radiology (María Carmen Aguilar-Hurtado and María Isabel Padin-Martín). Radiological progression was defined as a ≥20% increase in the presence and extension of ground-glass opacities, reticulation, honeycombing, diminished attenuation, centrilobular nodules, other nodules, emphysema, or consolidation compared with the HRCT scan at v0. Discrepancies in the reports were resolved by agreement. Data were collected at v0, v24, and v60 or the last lung and joint assessment if the patient did not reach v60. Data on adverse events were collected systematically at each visit by asking the patient about possible adverse events and infections they had. Adverse events were recorded in the medical history at each visit.

### 2.4. Working Definitions and Endpoints

The main endpoint was a composite endpoint, namely, “Progression to ILD at the end of follow-up” in terms of the following outcomes: (1) improvement (i.e., improvement in FVC ≥10% or DLCO ≥15% and absence of radiological progression); (2) nonprogression (stabilization or improvement in FVC ≤10% or DLCO <15% and absence of radiological progression); (3) progression (worsening of FVC >10% or DLCO >15% and radiological progression); or (4) death [30]. Radiological progression was defined as a ≥20% increase in the presence and extension of ground-glass opacities, reticulation, honeycombing, diminished attenuation, centrilobular nodules, other nodules, emphysema, or consolidation compared with the HRCT scan at v0.

The different patterns of ILD were defined based on the lung biopsy or HRCT according to the standard criteria of the American Thoracic Society/European Respiratory Society International Multidisciplinary Consensus Classification of the Idiopathic Interstitial Pneumonias [35], as follows: (1) nonspecific interstitial pneumonia (NSIP); (2) usual interstitial pneumonia (UIP); and (3) other (bronchiolitis obliterans, organizing pneumonia, lymphocytic interstitial pneumonitis, and mixed patterns). PFT included spirometry, whose results were expressed as percent predicted and adjusted for age, sex, and height. FVC was considered abnormal when <80% predicted. DLCO was evaluated using the single-breath method (DLCO-SB), and a value <80% was considered abnormal.

Other variables included the duration of joint symptoms, diagnostic delay, smoking history (current or past), body mass index (weight/height squared), Sjögren syndrome, and osteoporosis. Joint involvement was evaluated based on the DAS28 (28-joint Disease Activity Score) and its components [36], acute-phase reactants, and physical functioning (Health Assessment Questionnaire) [37]. We also collected variables associated with severity, as follows: rheumatoid factor (reference value, 20 U/mL; high titers, >60 U/mL); anticyclic citrullinated protein antibody (ACPA) (reference value, 10 U/mL, high values >340 U/mL); presence of at least 1 radiological erosion. We recorded treatment with csDMARDs, bDMARDs, immunosuppressants, and antifibrotic agents during follow-up before we also recorded the mean number of corticosteroids used during follow-up. Adverse effects were classified as mild and severe: mild adverse effects were easily tolerated signs and symptoms that do not require medical intervention or treatment; severe adverse events were those that required intervention, resulted in death, were life-threatening, required hospitalization or prolonged an existing hospitalization, led to a congenital abnormality, or led to significant disability [38].

### 2.5. Statistical Analysis

A descriptive analysis of the main variables was performed. Qualitative variables were expressed as whole numbers and percentages; quantitative variables were expressed as mean and standard deviation (SD) or as the median and interquartile range (IQR) depending on the normality of their distribution, as assessed using the Kolmogorov–Smirnov test. The bivariate analysis was performed using the paired *t* test or Wilcoxon test, as applicable, between v0 and the end of follow-up. The Kaplan–Meier and log-rank tests were used to estimate the survival of patients with RA-ILD and to compare survival between patients with the UIP and NSIP patterns. Survival time was measured from v0 until the end of follow-up (v60) or progression/death and type of censoring done was right censoring. Cox regression analysis was used to identify prognostic factors for the time to progression or death using univariate and multivariate models (forward stepwise). All variables reaching a *p* value of <0.10 were included in the Cox multivariate model. The incidence rates for total, severe, and mild adverse effects were also analyzed. The analysis was carried out using Rcommander.

## 3. Results

### 3.1. Baseline Clinical Characteristics

From March 2015 until December 2020, we prospectively followed up 116 patients with RA-ILD treated with DMARDs for a mean (SD) of 49.1 (14.4) months, that is, 454.9 patient-years. The main baseline characteristics are shown in Table 1. Patients were aged around 70 years, with an even distribution between the sexes. Half of the patients had been smokers or were smokers at inclusion. Almost all patients had long-term erosive and seropositive joint disease and, upon entering the study, they had had ILD for a mean of 2.2 years.

At v0, all patients were taking a DMARD: most were receiving a csDMARD and almost half were receiving a bDMARD. Fifty-nine patients (50.9%) were receiving a csDMARD in monotherapy, 36 (31%) were receiving a combination of a csDMARD and a bDMARD, 9 (7.8%) were receiving monotherapy with a bDMARD, 6 (5.1%) were receiving a combination of a csDMARD and an immunosuppressant, and a further 5 (4.3%) were taking a bDMARD with an immunosuppressant. Only one patient (0.9%) was receiving a csDMARD combined with nintedanib. The different DMARDs prescribed at v0 are shown in Table 1. More than half of the patients were taking corticosteroids. Eighty-four patients (72.4%) had received at least one csDMARD before v0, 29 (25%) had taken a bDMARD for a median (25%–75%) of 26 months (12.0–39.0), and 8 (6.8%) had received an immunosuppressant (Supplementary Table S1).

The most frequent radiological pattern was UIP in 71/116 patients (61.2%), followed by NSIP in 32/116 patients (27.6%), fibrotic NSIP in 9/116 patients (7.8), and other types of ILD in 4/116 patients (3.4%). UIP was confirmed by histopathology in 4 patients. Table 2 shows the differences in baseline characteristics between the UIP and NSIP patterns. Patients with the UIP pattern were more frequently male (*p* = 0.003) with positive ACPA titers (*p* = 0.020) and erosive disease (*p* = 0.023) than patients with NSIP. There was no difference between the groups in the remaining baseline clinical characteristics or in the treatment received. Summary statistics for all continuous variables are shown in (Supplementary Table S2).

### 3.2. Course of ILD after 60 Months of Follow-Up

**Figure 1:**
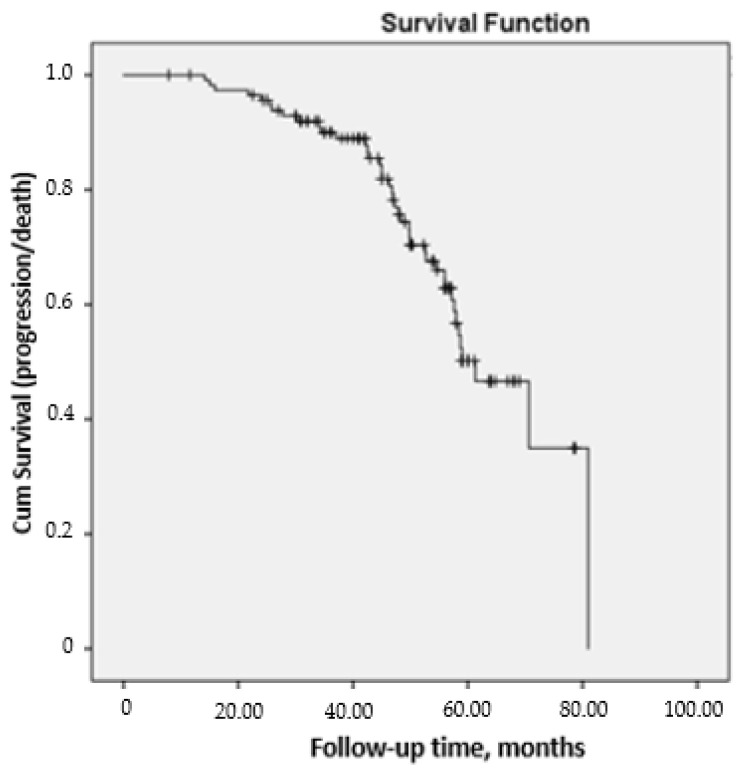
Survival curve was measured from v0 until the end of follow-up (v60) or progression/death in 116 patients at risk with RA-ILD under treatment with DMARDs.

**Figure 2:**
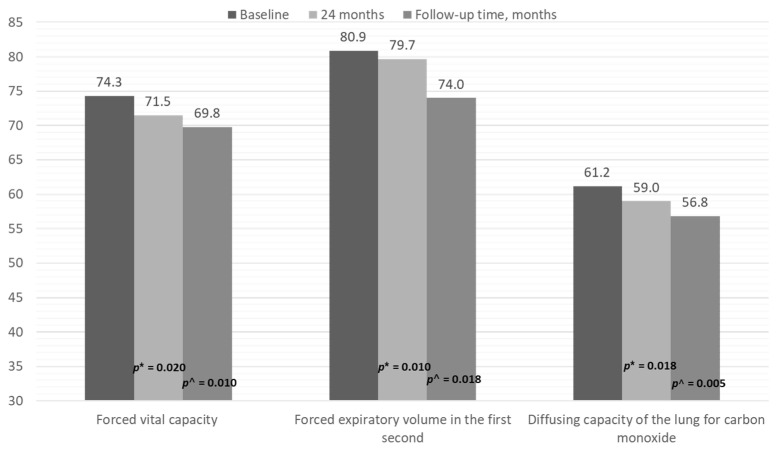
Progression of pulmonary function test results at the end of follow-up in patients with RA and ILD receiving DMARDs. * *p*-value for the comparison between 24 months and baseline; ^ *p*-value for the comparison between end of follow-up and baseline.

After 60 months of follow-up, a total of 98 patients (84.5%) remained in the study, 23 (19.8%) had progressed, and 18 (15.5%) had died, with a mean survival of 71.8 (1.9) months (Figure 1). As shown in Table 3, all mean PFT values were significantly worse at the end of follow-up. Figure 2 shows the progressive decrease in FVC, FEV_1_, and DLCO at 24 months and at the end of follow-up. HRCT showed that disease had progressed in 32/116 patients (27%), whereas 23/116 (20%) fulfilled the criteria for ILD and 18 (15%) died.

**Figure 3:**
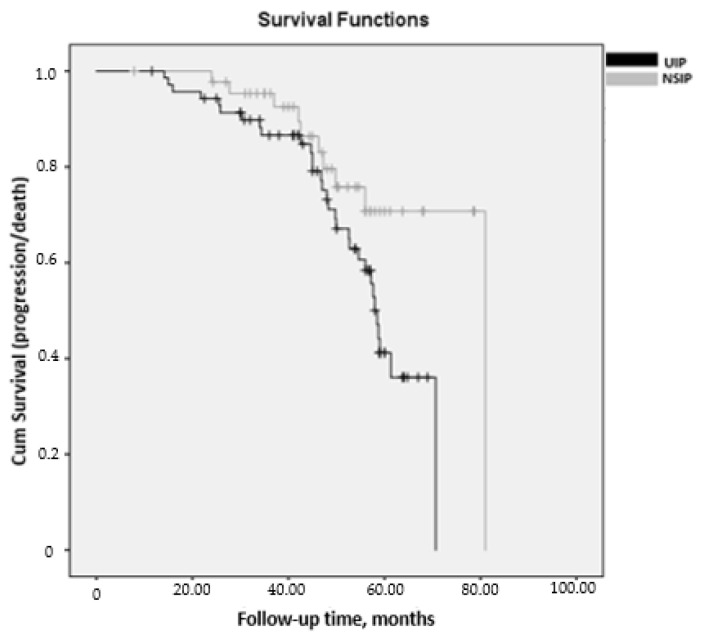
Kaplan–Meier survival curves measured from v0 until the end of follow-up (v60) or progression/death, stratified by radiological pattern in 116 patients at risk with RA-ILD. Survival was greater in patients with NSIP than in those with UIP (mean (95% CI), 70.0 months (63.5–76.5) vs. 55.7 months (51.6–59.8); *p* = 0.033, log-rank).

Table 4 shows the comparison between the UIP and NSIP patterns in the course of lung disease in patients with RA-ILD. The disease worsened more markedly in patients with the UIP pattern than in those with the NSIP pattern in terms of PFT values at the end of follow-up, especially in FVC (mean (SD) = 67.6 (22.6) vs. 78.6 (25.4); *p* = 0.037), with an incidence rate for progression (95% CI) of 0.11 (0.07–0.15) patient-years in those with UIP and 0.05 (0.02–0.09) patient-years in those with NSIP. Similarly, HRCT revealed a higher percentage of patients with disease progression (*p* = 0.002), overall progression of lung disease (*p* = 0.032), and mortality (*p* = 0.032). Survival was greater in patients with NSIP than in those with UIP (mean (95% CI), 70.0 months (63.5–76.5) vs. 55.7 months (51.6–59.8); *p* = 0.033, log-rank) (Figure 3).

With respect to joint involvement, patients remained stable in the last evaluation. At the end of follow-up, 52 patients were taking monotherapy with csDMARDs, 30 were taking combination therapy, 6 were taking monotherapy with bDMARDs, 5 were taking a csDMARD and an immunosuppressant, 4 were taking a bDMARD and an immunosuppressant, and 1 was taking an antifibrotic agent and mycophenolate. Twelve csDMARDs were modified (10 because of inefficacy and 2 because of adverse effects), and several bDMARDs were suspended for the following reasons: anti-TNF owing to progression of lung disease, 5 patients; tocilizumab owing to adverse effects, 2 patients; abatacept owing to ineffective treatment of joint involvement, 2 patients; and rituximab owing to adverse effects, 2 patients. Four patients started abatacept, 4 rituximab, and 1 started mycophenolate with an antifibrotic.

### 3.3. Factors Associated with Progression and Mortality of ILD after 60 Months of Follow-Up

**Figure 4:**
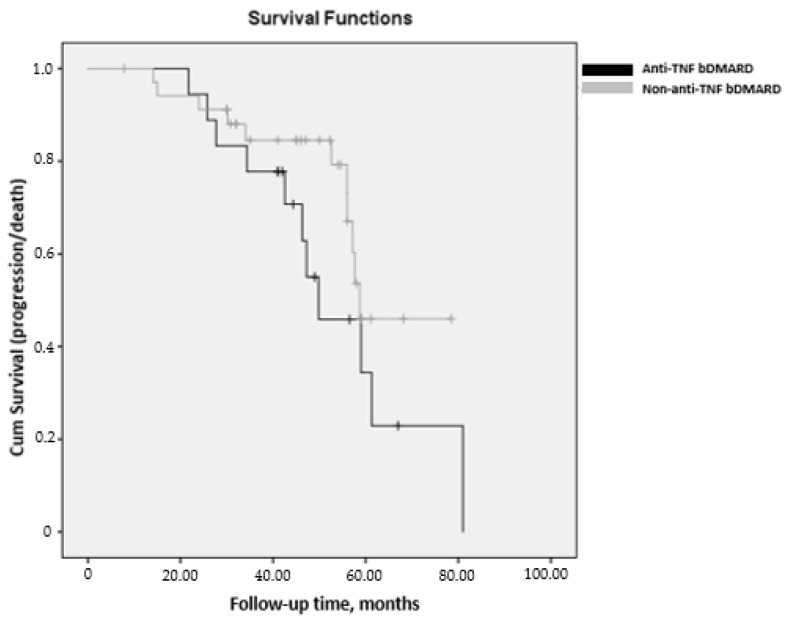
Kaplan–Meier survival curves measured from v0 until the end of follow-up (v60) or progression/death, stratified by bDMARD in 116 patients at risk with RA-ILD. Survival was greater for patients taking non–anti-TNF bDMARDs than for those taking anti-TNF bDMARDs (mean (95% CI), 62.7 months (57.3–68.1) vs. 54.5 months (43.8–65.2); *p* = 0.190, log-rank).

Table 5 shows the results of the Cox multivariate analysis (DV: progression or death), with a total of 116 patients with RA-ILD over a mean (SD) follow-up period of 49.1 (14.4) months. The event “progression” or “death” was recorded in 41/116 patients. The parameters assessed at baseline are included in the multivariate models. The multivariate analysis identified treatment with a non-anti-TNF bDMARD (i.e., abatacept, rituximab, or tocilizumab) as being associated with a 50% reduced risk of progression of ILD in patients with RA, whereas smoking, UIP radiological pattern, ACPA at high titers (>340), and FVC <80% at the initiation of follow-up were associated with a higher probability of progression of lung disease (Table 5). Survival was greater in patients with non–anti-TNF bDMARD than in those with anti-TNF bDMARD (mean (95%CI), 62.7 months (57.3–68.1) vs. 54.5 months (43.8–65.2); *p* = 0.190, log-rank) (Figure 4). The comparison of patients with RA-ILD according to bDMARD (anti-TNF or non-anti-TNF) is shown in Supplementary Table S3.

### 3.4. Adverse Events

The main adverse events are shown in Table 6. During follow-up, 79 patients (68.1%) had 88 adverse events, which were mainly mild (41.4%). Infection was the most common, affecting 71/116 patients (61%), especially respiratory infection (54.3%). The most severe events included eight (7.7%) that were not associated with infection: three tumors and five patients with rapid progression of ILD who died.

Supplementary Table S4 shows the follow-up times, treatment administered, and cause of death for the 18 (15.5%) patients who died. In addition, of the patients who died, only two discontinued DMARDs permanently: one with methotrexate and another with leflunomide owing to severe lung infection.

## 4. Discussion

We prospectively evaluated 116 patients with RA-ILD receiving treatment with various DMARDs after 60 months of follow-up. While PFT values fell slowly and progressively during follow-up, slightly more than one-third of the patients progressed poorly (20% experienced progression of lung disease and 15% died). According to many studies, most patients’ condition stabilizes or progresses slowly, although some patients progress quickly and die [33,39,40,41,42].

Previous studies have tried to identify factors associated with more marked progression and death in RA-ILD and specifically examine the effect of DMARDs on disease progression. In this sense, we did not find csDMARDs to be associated with a more pronounced progression of lung disease or death after 60 months of follow-up. Recent studies show that while methotrexate can lead to hypersensitivity pneumonitis during the first months of treatment [43], it was not associated with a greater risk of RA-ILD than in patients who do not take methotrexate [25]. Similarly, the results of a meta-analysis point to a lower risk of respiratory adverse events with leflunomide than with methotrexate or placebo [44]. Furthermore, bDMARDs have also been reported to trigger ILD, worsen existing ILD, and increase susceptibility to infection [45]. Our Cox multivariate analysis adjusted for follow-up time showed that non–anti-TNF bDMARDs were associated with a lower risk of progression of lung disease and mortality. In this sense, our group previously found that non-anti-TNF bDMARDs were associated with reduced progression of lung disease in patients with RA-ILD in the short term in a prospective cohort of 70 patients [33], although we observed that despite the increase in follow-up time and number of patients included, this association remains unchanged. We do not know whether these biologics have an intrinsic effect on RA-ILD, although studies published in the last few years point to the stabilization of lung disease with non-anti-TNF bDMARDs. Fernández-Díaz et al. [31] recently reported that lung disease remained stable or improved in 80% of patients with RA-ILD treated with abatacept. Abatacept has shown a more favorable effect on lung involvement in patients with RA-ILD than anti-TNF agents, as in the study by Nakashita et al. [46]. As for rituximab, we found that two of 17 patients who had been taking rituximab during follow-up died. While this drug is usually introduced in more severely ill patients and there may be an indication bias, rituximab was not associated with greater mortality or disease progression in these patients. Narvaez et al. [28] recently reported that rituximab can prove effective as rescue therapy in up to 80% of patients with progressive RA-ILD and major impairment of lung function. In the case of tocilizumab, some authors have reported isolated case reports of progression of RA-ILD [47,48], whereas others reported stabilization [32].

The multivariate analysis showed that some clinical–epidemiological factors such as smoking and high ACPA titers, as well as pulmonary factors such as the UIP radiological pattern and baseline FVC <80%, were associated with poorer pulmonary outcomes at the end of follow-up. A retrospective registry study of 290 patients with RA showed that smoking doubled the risk of ILD and that most patients had positive ACPA titers [40]. The lung damage caused by tobacco smoke and other harmful substances stimulates the citrullination of proteins associated with a break in immune tolerance and more severe RA [49]. As for PFT parameters, we found that the UIP radiological pattern was associated with a 2.5-fold greater risk of progression or death than the NSIP pattern. Furthermore, we were able to verify that the progression of lung disease was observed both in PFT and HRCT and that mortality was greater with lower survival than in patients with NSIP. This observation is consistent with findings from other studies, which show that patients with RA and the UIP pattern have an almost 3-fold greater risk of progression [50,51] and an almost 3-fold greater risk of dying than patients with the NSIP pattern [11]. However, it is noteworthy that only two patients in our study were treated with nintedanib, even though more than half had UIP despite the encouraging data on treatment of pulmonary fibrosis in the recent IMBUILD study [24], thus indicating that insufficient time had passed for the results to be translated to clinical practice in the same way as in idiopathic pulmonary fibrosis.

The present study has both limitations and strengths. First, patients with RA-ILD are prevalent cases that were already treated at initiation of follow-up, thus hampering interpretation of the effect of each drug during the natural course of ILD. The major drawback of including prevalent cases is that patients with stable ILD can be mixed with patients who have early-onset, progressive disease. However, the main objective of this prospective cohort study was to evaluate the course of lung and joint involvement in different types of patients with RA-ILD and treated under conditions of daily clinical practice. One of the strengths of this study is its 60-month prospective evaluation of patients with RA-ILD based on a comparison of various treatment strategies and clinical characteristics. Furthermore, there is no standard definition of progression of ILD, with some disparity between the criteria applied in the different studies we analyzed. However, we evaluated a concept of progression used in other studies of RA-ILD, that is, based on both PFT and HRCT parameters. Similarly, we evaluated the progression of each of these functional tests independently. Compared with other RA cohorts, the mean age of patients in our study was relatively high. This may be due to the fact that older age and late onset of the disease have been associated with an increased risk of RA-ILD [49,50]. However, the possibility of differences resulting from the inclusion of older patients should be considered. In relation to AEs, it may be that the definition used in our study of AEs, used in clinical trials (GCP guidelines), is responsible for finding lower rates of AEs compared to other prospective studies. The cox regression analysis and the analysis in two groups of bDMARDS were not adjusted based on matching using the propensity score or some multivariate method, so this may lead to a selection bias. Lastly, as this was a multicenter study, there could be differences in the evaluation of lung disease. In order to palliate this discrepancy, HRCT was centralized, thus enabling us to take advantage of the fact that radiology findings could be accessed remotely. In addition, thanks to our prospective follow-up, there were no missing data.

In conclusion, lung function stabilized, and inflammatory activity remained well controlled after 60 months of follow-up in more than half of patients with RA-ILD in treatment with various DMARDs. However, one-third of patients progressed quickly and died. csDMARDs were not associated with a significant risk of progression of lung disease. Among bDMARDs, anti-TNF agents were associated with risk of progression, while the non-anti-TNF bDMARDs are associated with a reduced risk of progression of lung disease. The main factors associated with progression of lung disease and death were smoking, high ACPA titers, lower FVC at baseline, and the UIP pattern. Identifying patients at greater risk of progression of lung disease will enable closer follow-up and more specific and earlier treatment.

## Figures and Tables

**Table 1:** Baseline characteristics of 116 with RA-ILD treated with DMARDs.

Variable	Total = 116
Epidemiological characteristics	
Female sex, *n* (%)	63 (54.3)
Caucasian race, *n* (%)	113 (97.4)
Age, years, mean (SD)	68.3 (9.9)
Clinical and analytical characteristics	
Current smoker	–
Nonsmoker, *n* (%)	57 (49.1)
Smoker, *n* (%)	23 (19.8)
Exsmoker, *n* (%)	36 (31.0)
Body mass index, mean (SD)	27.8 (4.1)
Time since diagnosis of RA, months, median (25%–75%)	148.5 (71.5–217.8)
Diagnostic delay, months, median (25%–75%)	8.5 (4.9–16.8)
Time since diagnosis of ILD, months, median (25%–75%)	27.5 (9.8–60.0)
Positive rheumatoid factor (>10), *n* (%)	111 (95.7)
Positive ACPA titer (>20), *n* (%)	100 (86.2)
High ACPA titer (>340), *n* (%)	48 (41.4)
Erosive disease, *n* (%)	76 (65.5)
Sjögren syndrome, *n* (%)	18 (15.5)
Osteoporosis, *n* (%)	51 (44.0)
Treatment	
Synthetic DMARD	100 (86.2)
Methotrexate, *n* (%)	51 (44.0)
Leflunomide, *n* (%)	30 (25.9)
Sulfasalazine, *n* (%)	9 (7.8)
Hydroxychloroquine, *n* (%)	21 (18.1)
Biologic DMARD	50 (43.1)
Infliximab, *n* (%)	1 (0.9)
Etanercept, *n* (%)	6 (5.2)
Adalimumab, *n* (%)	3 (2.6)
Golimumab, *n* (%)	3 (2.6)
Certolizumab, *n* (%)	3 (2.6)
Tocilizumab, *n* (%)	6 (5.2)
Abatacept, *n* (%)	15 (12.9)
Rituximab, *n* (%)	13 (11.2)
Immunosuppressants	11 (9.5)
Mycophenolate, *n* (%)	7 (6.0)
Azathioprine, *n* (%)	4 (3.4)
Antifibrotic agents, nintedanib, *n* (%)	1 (0.9)
Baseline corticosteroids, *n* (%)	69 (60.0)
Dose of baseline corticosteroids, median (25%–75%)	5.0 (0.0–7.5)

SD: standard deviation; RA: rheumatoid arthritis; ILD: interstitial lung disease; ACPA: anticyclic citrullinated protein antibody; DMARD: disease-modifying antirheumatic drug.

**Table 2:** Comparison of baseline characteristics between patients with RA-ILD according to radiological pattern (UIP and NSIP).

Variable	UIP, *n* = 71	NSIP, *n* = 41	*p* Value
Epidemiological characteristics			
Female sex, *n* (%)	31 (43.7)	30 (73.2)	0.003
Caucasian, *n* (%)	68 (95.8)	41 (100)	0.182
Age, years, mean (SD)	68.9 (9.4)	68.0 (10.9)	0.639
Clinical and analytical characteristics			
Current smoker			0.815
Nonsmoker, *n* (%)	32 (45.1)	21 (51.2)	
Smoker, *n* (%)	13 (18.3)	7 (17.1)	
Exsmoker, *n* (%)	26 (36.6)	13 (31.7)	
Body mass index, mean (SD)	28.1 (4.3)	27.5 (4.1)	0.578
Time since diagnosis of RA, months, median (25%–75%)	146.1 (69.2–227.9)	167.7 (87.5–224.2)	0.987
Diagnostic delay, months, median (25%–75%)	10.9 (4.9–18.4)	7.0 (4.9–15.5)	0.395
Time since diagnosis of ILD, months, mean (SD)	23.8 (9.6–59.9)	36.4 (11.3–67.9)	0.337
Positive rheumatoid factor (>10), *n* (%)	69 (97.2)	38 (92.7)	0.267
ACPA titer (>20), *n* (%)	65 (91.5)	31 (75.6)	0.020
Erosive disease, *n* (%)	53 (74.6)	22 (53.7)	0.023
Sjögren syndrome, *n* (%)	11 (15.5)	7 (17.1)	0.826
Osteoporosis, *n* (%)	32 (45.1)	17 (41.5)	0.711
Treatment			
Synthetic DMARD	60 (84.5)	37 (90.2)	0.390
Methotrexate, *n* (%)	28 (39.4)	22 (50.7)	0.145
Leflunomide, *n* (%)	20 (28.2)	9 (22.0)	0.469
Sulfasalazine, *n* (%)	7 (9.9)	1 (2.4)	0.142
Hydroxychloroquine, *n* (%)	14 (19.7)	7 (17.1)	0.730
Biologic DMARD	30 (42.3)	19 (46.3)	0.674
Infliximab, *n* (%)	1 (1.4)	0 (0.0)	0.445
Etanercept, *n* (%)	3 (4.2)	3 (4.2)	0.485
Adalimumab, *n* (%)	1 (1.4)	2 (4.9)	0.273
Golimumab, *n* (%)	2 (2.8)	1 (2.4)	0.905
Certolizumab, *n* (%)	2 (2.8)	1 (2.4)	0.905
Tocilizumab, *n* (%)	4 (4.2)	1 (2.4)	0.324
Abatacept, *n* (%)	9 (12.7)	6 (14.6)	0.769
Rituximab, *n* (%)	9 (12.7)	4 (9.8)	0.642
Immunosuppressants	7 (9.9)	4 (9.8)	0.986
Mycophenolate, *n* (%)	5 (7.0)	2 (4.9)	0.649
Azathioprine, *n* (%)	2 (2.8)	2 (4.9)	0.571
Antifibrotic agents, nintedanib, *n* (%)	1 (0.9)	0 (0.0)	0.045
Corticosteroids at baseline, *n* (%)	42 (59.1)	22 (50.7)	0.800
Dose of corticosteroids at baseline, median (25%–75%)	5.0 (0.0–6.0)	5.0 (0.0–7.5)	0.140

RA: rheumatoid arthritis; ILD: interstitial lung disease; DMARD: disease-modifying antirheumatic drug; SD: standard deviation; ACPA, anticyclic citrullinated protein antibody.

**Table 3:** Progress of symptoms and lung function at the end of follow-up of 116 patients with RA-ILS taking DMARDs.

Variable	Baseline	End of Follow-Up	*p* Value
Duration of follow-up, mean (SD)	–	49.1 (14.4)	–
Respiratory function			
Oxygen saturation, mean (SD)	96.1 (2.2)	95.0 (3.1)	0.018
Pulmonary function testing			
FVC, mean (SD)	74.3 (17.3)	69.8 (23.4)	0.010
FVC <80%, *n* (%)	69 (59.5)	79 (68.1)	0.012
FVC ≥80%, *n* (%)	47 (40.5)	37 (31.9)	
FEV_1_, mean (SD)	80.9 (20.5)	74.0 (21.1)	0.018
DLCO-SB, mean (SD)	61.2 (16.2)	56.8 (18.5)	0.005
HRCT			
Radiological pattern			0.720
UIP, *n* (%)	71 (61.2)	74 (63.7)	
NSIP, *n* (%)	32 (27.6)	31 (26.7)	
Fibrotic NSIP, *n* (%)	9 (7.8)	7 (6.0)	
Other, *n* (%)	4 (3.4)	4 (3.4)	
Course			
Progression, *n* (%)	–	32 (27.6)	
Stabilization, *n* (%)	–	77 (66.4)	
Improvement, *n* (%)	–	7 (6.0)	
Progression of lung disease (total) *			
Improvement, *n* (%)	–	9 (7.8)	
Stabilization, *n* (%)	–	66 (56.9)	
Worsening, *n* (%)	–	23 (19.8)	
Death, *n* (%)	–	18 (15.5)	
Inflammatory activity			
DAS28, median (25%–75%)	2.8 (2.3–4.0)	3.0 (3.0–5.2)	0.627
C-reactive protein, median (25%–75%)	5.3 (2.9–13.0)	8.0 (2.0–22.0)	0.320
ESR, median (25%–75%)	21.0 (9.7–36.5)	20.0 (8.0–29.0)	0.136
HAQ, median (25%–75%)	1.0 (0.2–1.8)	1.1 (0.6–1.9)	0.484

RA: rheumatoid arthritis; ILD: interstitial lung disease; DMARD: disease-modifying antirheumatic drug; SD: standard deviation; FVC: forced vital capacity; FEV_1_: forced expiratory volume in the first second; DLCO: diffusing capacity of the lung for carbon monoxide; UIP: usual interstitial pneumonia; NSIP: nonspecific interstitial pneumonia; HRCT: high-resolution computed tomography; DAS28: 28-joint Disease Activity Score; ESR: erythrocyte sedimentation rate; HAQ: Health Assessment Questionnaire; * Progression of lung disease (total): taking into account HRCT and pulmonary function testing (FVC and DLCO).

**Table 4:** Comparison of progression of symptoms and lung disease in patients with RA-ILD according to radiological pattern (UIP or NSIP).

Variable	UIP	NSIP	*p* Value
Duration of follow-up, mean (SD)	46.8 (14.5)	47.5 (14.9)	0.808
Pulmonary function testing			
Last FVC, mean (SD)	67.6 (22.0)	78.6 (25.4)	0.037
Last FEV_1_<, mean (SD)	72.1 (21.9)	78.2 (20.7)	0.225
Last DLCO, mean (SD)	66.9 (18.5)	70.1 (16.7)	0.235
HRCT			
Course			0.002
Progression, *n* (%)	27 (38.0)	9 (22.0)	
Stabilization, *n* (%)	44 (62.0)	26 (63.4)	
Improvement, *n* (%)	0 (0.0)	6 (14.6)	
Progression of lung disease (total) *			0.032
Improvement, *n* (%)	2 (2.8)	6 (14.6)	
Stabilization, *n* (%)	38 (53.5)	25 (61.0)	
Worsening, *n* (%)	16 (22.5)	7 (17.1)	
Death, *n* (%)	15 (21.1)	3 (7.3)	
Inflammatory activity			
Last DAS28, mean (SD)	3.3 (1.1)	3.2 (1.4)	0.428
Last C-reactive protein, mean (SD)	18.5 (16.9)	8.6 (7.3)	0.037
Last ESR, mean (SD)	26.1 (17.4)	23.2 (18.2)	0.823
Last HAQ, mean (SD)	1.5 (0.7)	1.2 (0.7)	0.341

RA: rheumatoid arthritis; ILD: interstitial lung disease; DMARD: disease-modifying antirheumatic drug; SD: standard deviation; FVC: forced vital capacity; FEV_1_: forced expiratory volume in the first second; DLCO: diffusing capacity of the lung for carbon monoxide; UIP: usual interstitial pneumonia; NSIP: nonspecific interstitial pneumonia; HRCT: high-resolution computed tomography; DAS28: 28-joint Disease Activity Score; ESR: erythrocyte sedimentation rate; HAQ: Health Assessment Questionnaire; * Progression of lung disease (total): taking into account HRCT and pulmonary function testing (FVC and DLCO).

**Table 5:** Multivariate analysis of progression and mortality of lung disease in patients with RA-ILD taking DMARDs.

Variable	Univariate HR (95% CI)	Multivariate HR (95% CI)	*p* Value
Age, years	1.930 (0.98–1.07)		
Male sex	1.041 (0.28–2.23)		
History of smoking	2.204 (1.01–4.85)	2.543 (1.03–6.24)	0.042
Radiological pattern, UIP	2.712 (1.82–7.11)	2.661 (1.04–6.77)	0.040
Rheumatoid factor, titer	1.001 (1.00–1.01)		
High ACPA (>340)	2.556 (1.17–5.58)	2.810 (1.17–6.75)	0.021
Baseline FVC <80	2.517 (1.10–5.75)	3.840 (1.50–6.70)	0.003
Baseline DLCO-SB <80	2.800 (0.90–8.10)		
Corticosteroids	1.603 (0.71–3.57)		
DMARDs	0.662 (0.22–1.93)		
Immunosuppressants	0.661 (0.16–2.64)		
Anti-TNF	2.692 (1.02–7.87)		
Non–anti-TNF	0.618 (0.36–0.89)	0.472 (0.25–0.86)	0.014

R^2^ = 0.316. RA: rheumatoid arthritis; ILD: interstitial lung disease; DMARD: disease-modifying antirheumatic drug; HR: hazard ratio. Independent variables: sex, age, history of smoking, radiological pattern (UIP/NSIP), baseline FVC, baseline DLCO-SB, tumor necrosis factor inhibitors (anti-TNF) treatment (infliximab, adalimumab, etanercept, golimumab, certolizumab), non-anti-TNF (rituximab, abatacept, tocilizumab), csDMARDs (methotrexate, leflunomide, hydroxychloroquine, sulfasalazine), immunosuppressants (azathioprine, mycophenolate), corticosteroids.

**Table 6:** Adverse effects in patients with RA-ILD taking DMARDs.

Variable	Sample = 116
Adverse effects, *n* (%)	79 (68.1)
Mild adverse effects, *n* (%)	48 (41.4)
Severe adverse effects, *n* (%)	40 (34.4)
Incidence of adverse effects (patient-years)	0.17
Incidence of mild adverse effects (patient-years)	0.10
Incidence of severe adverse effects (patient-years)	0.08
Infection, *n* (%)	71 (61.2)
Respiratory infection, *n* (%)	63 (54.3)
Other infections, *n* (%)	13 (11.2)
Cold sore, *n* (%)	2 (1.7)
Dental infection, *n* (%)	1 (0.8)
Cutaneous infection, *n* (%)	3 (2.5)
Urinary infection, *n* (%)	7 (6.0)
Incidence of infection (patient-years)	0.15
Incidence of respiratory infection (patient-years)	0.13
Incidence of other infections (patient-years)	0.02
Mortality	18 (15.5)
Incidence of mortality (patient-years)	0.03

RA: rheumatoid arthritis; ILD: interstitial lung disease. Patient-years: incidence per patient-years during observation time in the study.

## Data Availability

Data presented in this study are available on request from the corresponding author.

## Supplementary Materials

The following are available online at https://www.mdpi.com/2077-0383/10/4/874/s1, Table S1: Previous treatment in patients with RA-ILD, Table S2: Summary statistics for all continuous variables in 116 patients with RA-ILD, Table S3: Comparison of patients with RA-ILD according to bDMARD (anti-TNF or non-anti-TNF) at the beginning of the observation period, Table S4: Characteristics of patients with RA-ILD taking DMARDs who died.
