# Supplementary material for: Predictors of Progression and Mortality in Patients with Prevalent Rheumatoid Arthritis and Interstitial Lung Disease: A Prospective Cohort Study
Source: J Clin Med. 2021 Feb 20;10(4):874. doi: 10.3390/jcm10040874 (PMC7924396; doi:10.3390/jcm10040874)
Supplement: Supplementary file 1 [file jcm-10-00874-s001.pdf]

Supplementary Table S1. Previous treatment in patients with RA-ILD.

Variable	Sample = 116
Synthetic DMARD	
Methotrexate, *n* (%)	63 (54.3)
Leflunomide, *n* (%)	40 (34.5)
Sulfasalazine, *n* (%)	26 (22.4)
Hydroxychloroquine, *n* (%)	27 (23.3)
Biologic DMARD	
Infliximab, *n* (%)	6 (5.2)
Etanercept, *n* (%)	6 (5.2)
Adalimumab, *n* (%)	6 (5.2)
Golimumab, *n* (%)	1 (1.9)
Certolizumab, *n* (%)	2 (1.7)
Tocilizumab, *n* (%)	6 (5.2)
Abatacept, *n* (%)	3 (2.6)
Rituximab, *n* (%)	4 (3.4)
Immunosuppressants	
Mycophenolate, *n* (%)	2 (1.7)
Azathioprine, *n* (%)	4 (3.4)
Cyclophosphamide, *n* (%)	8 (6.9)

Abbreviations. RA: rheumatoid arthritis; ILD: interstitial lung disease; DMARD: disease-modifying antirheumatic drug.

Supplementary Table S2. Summary statistics for all continuous variables in 116 patients with RA-ILD.

Variable	Mean	SD	Min	Max	P25	P50	P75
Age, years	68.3	9.9	42.1	87.1	62.3	70.0	75.3
Body mass index	27.8	4.1	19.2	40.8	25.2	27.9	30.0
Time since diagnosis of RA, months	170.8	120.1	4.2	400.3	71.5	148.5	217.8
Diagnostic delay, months	14.8	16.8	0.0	60.1	4.9	8.5	16.8
Time since diagnosis of ILD, months	36.4	30.7	0.0	126.0	9.8	27.5	60.0
FVC, baseline	74.3	17.3	33	121	62.0	74.0	88.0
FEV1, baseline	80.9	20.5	40	192	65.0	78.5	94.0
DLCO-SB, baseline	61.2	16.2	23	99	48.5	59.5	69.0
FVC, end of follow-up	69.8	23.4	31	132	55.5	70.0	87.5
FEV1, end of follow-up	74.0	21.1	35	124	58.0	76.0	91.0
DLCO-SB, end of follow-up	56.8	18.5	18	116	43.0	56.0	70.5
DAS28, baseline	3.2	1.2	0.9	7.0	2.3	2.8	4.0
DAS28, end of follow-up	3.7	1.5	0.7	6.0	2.9	3.0	5.2
HAQ, baseline	1.0	1.1	0.0	3.0	0.2	1.0	1.8
DAS28, end of follow-up	1.2	0.8	0.0	3.0	0.6	1.1	1.9

Abbreviations. RA: rheumatoid arthritis; ILD: interstitial lung disease; SD: standard deviation; FVC: forced vital capacity; FEV_1: forced expiratory volume in the first second; DLCO: diffusing capacity of the lung for carbon monoxide.

Supplementary Table S3. Comparison of patients with RA-ILD according to bDMARD (anti-TNF or non-anti-TNF) at the beginning of the observation period.

Variable	Anti-TNF *n* = 16	Non-anti-TNF *n* = 34	*p*-Value
Epidemiological characteristics			
Female sex, *n* (%)	14 (87.5)	15 (44.1)	0.010
Caucasian race, *n* (%)	16 (100.0)	33 (97.1)	0.488
Age, years, mean (SD)	67.6 (12.4)	65.6 (10.0)	0.544
Clinical and analytical characteristics			
Current smoker			0.867
Nonsmoker, *n* (%)	9 (56.3)	17 (50.0)	
Smoker, *n* (%)	3 (18.8)	6 (17.6)	
Exsmoker, *n* (%)	4 (25.0)	11 (32.4)	
Body mass index, mean (SD)	29.3 (4.2)	27.4 (4.2)	0.211
Time since diagnosis of RA, months, mean (SD)	250.6 (125.6)	182.8 (117.2)	0.060
Diagnostic delay, months, mean (DS)	20.7 (18.6)	19.9 (16.4)	0.898
Time since diagnosis of ILD, months, mean (SD)	55.0 (35.9)	41.1 (32.6)	0.182
Positive rheumatoid factor (>10), *n* (%)	16 (100)	33 (97.1)	0.488
Positive ACPA titer (>20), *n* (%)	15 (93.8)	32 (94.1)	0.959
Erosive disease, *n* (%)	12 (75.0)	25 (73.5)	0.912
Synthetic DMARD	14 (87.5)	24 (70.6)	0.192
Corticosteroids at baseline, *n* (%)	10 (62.5)	21 (61.8)	0.960
DAS28, mean (SD)	3.1 (1.4)	3.2 (1.2)	0.754
HAQ, mean (SD)	0.9 (0.7)	1.3 (0.8)	0.157
Radiological pattern			
UIP, *n* (%)	9 (56.3)	20 (58.8)	0.863
NSIP, *n* (%)	7 (43.8)	14 (41.2)	0.863
Respiratory function			
FVC, mean (SD)	72.7 (15.5)	80.0 (16.7)	0.185
FEV_1, mean (SD)	89.6 (33.2)	82.9 (16.5)	0.389
DLCO-SB, mean (SD)	58.0 (6.3)	64.6 (15.0)	0.180

Progression of lung disease (total)*			0.033
Improvement/Stabilization, *n* (%)	7 (43.8)	24 (70.6)	
Worsening/Death, *n* (%)	9 (56.3)	10 (29.4)	

Abbreviations. RA: rheumatoid arthritis; ILD: interstitial lung disease; DMARD: disease-modifying antirheumatic drug; SD: standard deviation; FVC: forced vital capacity; FEV_1: forced expiratory volume in the first second; DLCO-SB: diffusing capacity of the lung for carbon monoxide (single breath); UIP: usual interstitial pneumonia; NSIP: nonspecific interstitial pneumonia.

Supplementary Table S4. Characteristics of patients with RA-ILD taking DMARDs who died.

Patient	Follow-up (months)	Treatment	Duration of Treatment (Months)	Cause of Death
Patient 1	62.5	Leflunomide	108.5	COVID-19
Patient 2	34.3	Methotrexate	179.3	Pulmonary adenocarcinoma
Patient 3	34.0	Rituximab	71.7	Progression of ILD and pulmonary superinfection
Patient 4	15.0	Etanercept and hydroxychloroquine	25.0	Progression of ILD and pulmonary superinfection
Patient 5	57.1	Rituximab and hydroxychloroquine	74.0	Progression of ILD
Patient 6	25.7	Infliximab and leflunomide	146.2	Progression of ILD
Patient 7	27.7	Etanercept	160.3	Progression of ILD and respiratory superinfection
Patient 8	46.8	Methotrexate and hydroxychloroquine	63.3	Progression of ILD and respiratory superinfection
Patient 9	48.3	Sulfasalazine	48.3	Progression of ILD and respiratory superinfection
Patient 10	52.6	Tocilizumab and methotrexate	64.1	Brain hemorrhage
Patient 11	55.9	Etanercept	60.1	Progression of ILD and respiratory superinfection
Patient 12	15.9	Nintedanib and leflunomide	10.5	Progression of ILD and respiratory superinfection
Patient 13	30.2	Methotrexate	99.8	Primary cerebral lymphoma
Patient 14	25.6	Sulfasalazine	33.3	Progression of ILD and respiratory superinfection
Patient 15	54.6	Hydroxychloroquine and mycophenolate	84.1	Respiratory and urinary sepsis
Patient 16	49.9	Methotrexate followed by leflunomide	24 followed by 22	Progression of ILD
Patient 17	14.1	Cyclophosphamide followed by mycophenolate	36	Progression of ILD
Patient 18	47.2	Adalimumab	120.5	Progression of ILD

Abbreviations. RA: rheumatoid arthritis; ILD: interstitial lung disease.
